# Women and HIV in the United States

**DOI:** 10.1371/journal.pone.0172367

**Published:** 2017-02-16

**Authors:** Alexander Breskin, Adaora A. Adimora, Daniel Westreich

**Affiliations:** 1 Department of Epidemiology, UNC-Chapel Hill, Chapel Hill, North Carolina, United States of America; 2 Department of Medicine, UNC-Chapel Hill, Chapel Hill, North Carolina, United States of America; Agencia de Salut Publica de Barcelona, SPAIN

## Abstract

**Background:**

The demographic and geographic characteristics of the HIV epidemic in the US has changed substantially since the disease emerged, with women in the South experiencing a particularly high HIV incidence. In this study, we identified and described counties in the US in which the prevalence of HIV is particularly high in women compared to men.

**Methods:**

Using data from AIDSVu, a public dataset of HIV cases in the US in 2012, we categorized counties by their decile of the ratio of female to male HIV prevalence. The demographic and socioeconomic characteristics of counties in the highest decile were compared to those of counties in the lower deciles.

**Results:**

Most of the counties in the highest decile were located in the Deep South. These counties had a lower median income, higher percentage of people in poverty, and lower percentage of people with a high school education. Additionally, people with HIV in these counties were more likely to be non-Hispanic black.

**Conclusions:**

Counties with the highest ratios of female-to-male HIV prevalence are concentrated in the Southern US, and residents of these counties tend to be of lower socioeconomic status. Identifying and describing these counties is important for developing public health interventions.

## Introduction

In the first decade of the HIV epidemic, disparities in HIV incidence became clear in the United States—racial and ethnic minorities and men who have sex with men in large, coastal cities were hardest hit, and the incidence rate in men was nearly fifteen times the rate in women.[1] By 2010, the characteristics of the HIV-infected population had shifted dramatically: women composed 21% of HIV cases in the United States and the incidence rate for men was only 3 times the rate in women.[2] Additionally, the geographic distribution of cases changed, particularly among women; women in southern states now have one of the highest incidence rates of HIV among women of all regions of the country.[2]

While recent studies have described the current demographic characteristics of HIV-infected people in different parts of the United States, there are none that directly present the characteristics of regions defined by high HIV prevalence among women compared with men. The purpose of this study is to identify and describe these regions.

## Methods

### Data sources

We analyzed data from AIDSVu [3], a free, public-use online resource describing the county-level prevalence of HIV in the United States in 2012. The methods of data collection and calculations for AIDSVu are described on the website (www.aidsvu.org). Briefly, AIDSVu comprises HIV surveillance data from state and local health departments that was organized by the US Centers for Disease Control and Prevention. AIDSVu also provides US Census Bureau estimates of economic and demographic variables. County-level population estimates for 2012 were also obtained from the US Census Bureau.[4]

### Exclusion criteria

Data were suppressed from the AIDSVu data set if certain criteria were met that would indicate the possibility of identifying individual cases, including having fewer than 5 HIV cases in a demographic category within a county (the full set of criteria are available on the AIDSVu website). For instance, if there were fewer than 5 female cases in a county, then cases categorized by sex would be suppressed for that county. Counties with a correctional facility were also excluded, as relatively high HIV prevalence among inmates likely distorts estimated HIV prevalence in such counties. Lastly, we raised the minimum number of HIV cases necessary for inclusion to 12 among either men or women as this would lead to instability in the prevalence, as defined by AIDSVu.[5]

### Characterization of counties

Counties were ranked by the female-to-male HIV prevalence ratio. We compared counties in the highest decile of the female:male ratio with counties in the lower nine deciles. The total populations of counties in each decile-based category of female:male prevalence ratio (highest decile, lower deciles), including both HIV cases and non-cases, were compared on the following variables: median income; Gini coefficient (a measure of population income inequality; higher values indicate greater inequality) [6]; race; age; sex; and percentage of the population: living in poverty, with at least a high school education, and without health insurance. We also computed the distribution of race and sex among HIV cases in each decile-based category. To understand the geographic distribution of these counties, we mapped the location of counties by decile category.

### Statistical methods

We computed estimates of the median income, average Gini coefficient, and frequency distributions of the remaining variables for each decile-based category weighted by the total population in the included counties. We computed confidence limits for all of the estimates using a non-parametric bootstrap, in which counties were selected with replacement and decile-categories were estimated within each replication.

### Sensitivity analysis

Due to the large number of counties with unstable prevalence estimates excluded from the main analysis, we conducted several sensitivity analyses to assess the impact of these exclusion criteria. First, we included the counties with unstable prevalence estimates by including counties with more than 5 but fewer than 12 HIV cases. Next, we used two methods to stabilize prevalence estimates: geographic-based and regression-based empirical Bayesian smoothing [7–9], both of which were applied to the male and female prevalence estimates separately in each county. The geographic-based smoothing was conducted in GeoDa.[10] Sets of counties near the target county (see below) were chosen, and a variance-weighted average prevalence from those counties was computed and set as the prior prevalence for the target county. A variance-weighted average of the prior prevalence and the observed prevalence of the target county was then assigned as the smoothed prevalence of the target county. Three methods were used in separate analyses to choose the set of counties used for smoothing: the 5 counties with geographical centers closest to the geographical center of the target county, all contiguous counties, and all counties within 100 miles of the target county. For the regression-based smoothing, a regression of the prevalence of HIV was fit against county demographic characteristics. The predicted prevalence for the target county from this regression was used as a Bayesian prior [7–9] for the prevalence. A variance-weighted average of the prior prevalence and the observed prevalence was then assigned as the smoothed prevalence of the target county.

## Results

**Fig 1:**
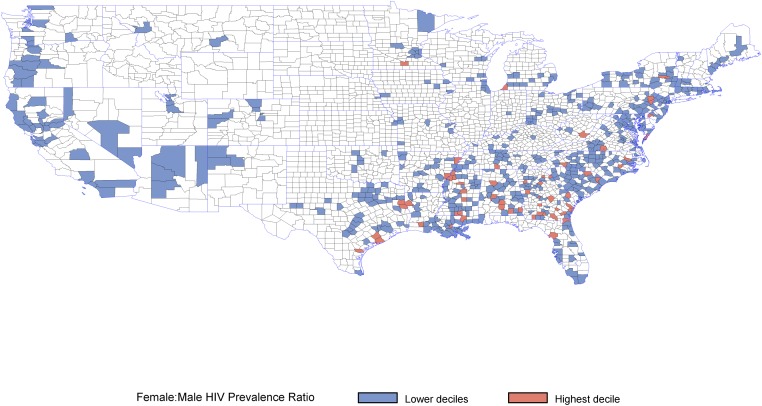
Counties with the top decile of female to male HIV prevalence ratio. Counties in white are excluded from the analysis due to the presence of a correctional facility or because data was suppressed by AIDSVu due to low (<5) HIV case counts among men or women.

After excluding counties with correctional facilities or unstable rates, 612 counties (out of 3,144 United States counties) were available for comparisons, which included 640,985 (72%) cases of HIV out of a total of 886,989 cases available in AIDSVu. Among 152,849 HIV-infected women (about 25% of cases included in this analysis), 2,375 (2%) lived in the 61 counties in the highest decile of the female:male HIV prevalence ratio, and 150,474 (98%) lived in the 551 counties in the lower deciles. Counties in the highest decile of the female:male HIV prevalence ratio are shown in Fig 1; these counties were largely concentrated in the Deep South.

The populations (HIV-positive and -negative) of counties in the highest decile had a higher percentage of people living in poverty, and lower percentage of people with a high school education. These counties also had a substantially higher proportion of both non-Hispanic white residents (69% vs. 58%) and non-Hispanic black residents (21% vs. 14%), and a lower proportion of residents of other race/ethnicities (3% vs. 9%) and residents of Hispanic ethnicity (7% vs. 19%) in their overall populations compared with counties with lower deciles.

Considering the 640,985 HIV cases included in the analysis, those living in the highest decile were more likely to be black than those living in other counties (58% vs. 42%). In the highest decile, nearly 1 in 2 cases of HIV were among women; in the lower deciles, only 1 in 4 cases of HIV were among women.

Additional comparisons of the total populations and HIV cases of the highest decile counties compared with other counties, as well as descriptive statistics describing the overall populations of counties excluded from the analysis, are shown in Table 1.

**Table 1:** Characteristics of counties (total population, and HIV cases only) by decile of female-to-male HIV prevalence ratio. All figures are given as % (95% CI) unless noted. There were 61 counties in the top decile and 551 in the remaining deciles.

Characteristics	Total population			HIV Cases^a^	
	Highest F:M Decile	Lower F:M Deciles	Excluded Counties^b^	Highest F:M Decile	Lower F:M Deciles
Income (Median, IQR)^c^	41.9 (36.2, 52.7)	52.2 (50.9, 52.9)	48.4 (47.0, 49.8)		
Gini Coefficient (Average, CI)	0.44 (0.43, 0.46)	0.46 (0.45, 0.47)	0.44 (0.44, 0.45)		
Living in poverty	18.9 (17.2, 21.6)	16.1 (15.3, 16.9)	15.8 (15.2, 16.4)		
HS Education or more	82.4 (79.6, 84.0)	85.4 (84.2, 86.0)	86.0 (85.2, 86.6)		
Uninsured	17.3 (15.9, 19.5)	17.5 (16.3, 18.6)	16.3 (15.6, 17.1)		
Female	51.4 (51.1, 51.6)	51.1 (51.0, 51.3)	50.5 (50.4, 50.6)	45.0 (43.3, 46.5)	23.7 (20.9, 26.6)
Race/Ethnicity					
White (non-Hispanic)	68.7 (63.3, 71.5)	57.9 (55.1, 61.9)	71.0 (69.4, 72.9)	26.9 (21.6, 31.4)	31.6 (27.2, 36.0)
Black (non-Hispanic)	20.5 (17.7, 26.0)	14.4 (12.8, 15.8)	9.7 (8.8, 10.5)	58.3 (49.0, 67.8)	41.8 (36.5, 47.0)
Hispanic	7.9 (5.4, 10.0)	19.1 (15.4, 21.5)	13.2 (11.4, 14.6)		
Other	2.9 (2.3, 3.2)	8.7 (7.4, 9.6)	6.2 (5.3, 6.7)		
Age					
0 to 14	20.1 (19.4, 21.2)	19.9 (19.5, 20.3)	19.7 (19.4, 20.0)		
15 to 24	13.7 (13.0, 14.5)	14.3 (14.1, 14.5)	13.9 (13.7, 14.2)		
25 to 34	11.6 (11.2, 12.3)	14.0 (13.6, 14.3)	12.5 (12.3, 12.7)		
35 to 44	12.8 (12.2, 13.5)	13.7 (13.5, 13.8)	12.9 (12.8, 13.0)		
45 to 54	14.9 (14.3, 15.2)	14.4 (14.3, 14.6)	14.8 (14.6, 14.9)		
55+	27.1 (25.0, 28.4)	23.7 (23.1, 24.3)	26.2 (25.8, 26.8)		

IQR, interquartile range; CI, 95% confidence interval

^a^48 counties excluded due to low case counts in race categories. Hispanic and other not shown due to low case counts

^b^ 2,532 counties excluded from analysis due to the presence of a correctional facility or because data was suppressed by AIDSVu due to low (<5) HIV case counts among men or women.

^c^Thousands of US dollars.

### Sensitivity analysis

Including additional counties with unstable rates and applying several different rate stabilization techniques did not qualitatively change the results (not shown). The counties excluded from the analysis had similar levels of socioeconomic variables as the counties in the lower deciles of the female:male HIV prevalence ratio. The excluded counties, however, had a higher proportion of non-Hispanic white residents and a lower proportion of non-Hispanic black residents compared with those included in the analysis.

## Discussion

In this study, we identified counties with the highest burden of HIV among women compared with men, and we provided a description of the socioeconomic and demographic characteristics of the overall populations of these counties. In contrast to prior studies, we identified these counties and then described their location, as opposed to dividing the United States into regions and then describing the burden of HIV among women in each region. Several socioeconomic and demographic characteristics are striking.

Counties in the highest decile of female:male HIV prevalence ratio were concentrated in the South, consistent with previous reports of the southern HIV epidemic.[2] These counties also had a higher proportion of people living in poverty and a higher proportion of people with less than a high school education. People living with HIV in this region are known to have worse access to care,[11] initiate treatment later,[12] and have worse survival than those in other regions of the United States.[2] Interestingly, the age distribution of the population of counties in the lower deciles slightly shifted toward younger individuals—perhaps reflecting the persistently higher incidence of HIV among young men who have sex with men.[13]

There are several limitations to this study. First, due to the public nature of this data, a substantial portion of the data was suppressed to prevent individuals from becoming identifiable. This suppression prevented large portions of the United States, particularly in the mid-western region, from being included in the analysis. However, the reason for suppression was due to low HIV case count, and thus it is unlikely that these regions would substantially change the results of the analysis—indeed, data from unsuppressed counties represented 72% of all persons living with HIV in the United States. Additionally, sensitivity analyses that included some otherwise excluded counties and applied stabilization methods to the prevalence estimates produced no meaningful difference in the results. Second, AIDSVu provides prevalence, not incidence, data. Prevalence is a function of the incidence of new HIV cases, in- and out-migration of HIV cases from the region, and the survival time of people living with HIV, and therefore it may not provide an accurate picture of new HIV cases, especially if survival time with HIV is associated with county characteristics.

Despite these limitations, our results are still useful for describing the burden of disease in these counties. Our publicly available data-driven approach confirmed previous findings, namely that socioeconomically disadvantaged counties in the southern United States face the highest burden of HIV among women compared with men. By identifying the counties with the highest prevalence of HIV among women compared with men, targeted interventions that are most appropriate for the unique characteristics of this population can be developed and implemented in the locations with the highest need, thus maximizing their effectiveness and impact.
